# Breast Cancer Presentation, Treatment Patterns, and Progression‐Free Survival at a Tertiary Oncology Centre in Bangladesh: A Retrospective Study

**DOI:** 10.1002/cnr2.70623

**Published:** 2026-07-10

**Authors:** Qamruzzaman Chowdhury, Md Arifur Rahman, Ferdous Ara Begum, Ayasha Shiddika, Md Nurunnabi, Sharmin Akter Rupa, Mashud Parvez, S. M. Khodeza Nahar Begum, Salma Sultana

**Affiliations:** ^1^ Department of Oncology Bangladesh Specialized Hospital Dhaka Bangladesh; ^2^ Department of Radiology and Imaging Bangladesh Specialized Hospital Dhaka Bangladesh; ^3^ Department of Pathology Bangladesh Specialized Hospital Dhaka Bangladesh; ^4^ Department of Surgery Bangladesh Specialized Hospital Dhaka Bangladesh

**Keywords:** Bangladesh, breast cancer, HER2‐positive, luminal A, molecular subtype, progression‐free survival, retrospective study, triple‐negative breast cancer

## Abstract

**Background:**

Breast cancer remains a major cause of cancer‐related morbidity among women worldwide, with persistent disparities in diagnosis, treatment access, and outcomes across low‐ and middle‐income settings. Institution‐based real‐world data are limited in Bangladesh. This study aimed to describe the clinicopathological characteristics, molecular subtype distribution, documented treatment patterns, and progression‐related outcomes of breast cancer patients managed at a tertiary care center in Bangladesh.

**Methods:**

This retrospective observational study was conducted at Bangladesh Specialized Hospital, Dhaka, Bangladesh. Hospital records of 1058 adult breast cancer patients were reviewed. Data on demographics, tumor characteristics, molecular subtype, documented treatment modalities, and follow‐up were analyzed. Progression‐free survival (PFS) was assessed using Kaplan–Meier methods and Cox proportional hazards regression.

**Results:**

Luminal A was the most common primary molecular subtype, accounting for 38.47% of cases, followed by TNBC at 23.53% and HER2‐positive disease at 11.91%. Documented metastatic or recurrent disease during the available records was present in 34.59% of patients, while Stage III or IV disease at diagnosis accounted for 33.65% of the cohort. Chemotherapy was the most frequently documented treatment modality, recorded in 79.02% of patients, followed by endocrine therapy in 53.78%, breast surgery in 56.52%, HER2‐directed therapy in 21.17%, and radiotherapy in 20.04%. Among luminal cases, endocrine therapy was documented in 86.61%, and among the HER2‐positive analytical subgroup, HER2‐directed therapy was documented in 85.96%. A total of 59 PFS events were recorded, comprising 58 progression events and one death. In multivariable Cox analysis, Luminal B, HER2‐negative disease, adjusted hazard ratio 2.97, 95% CI 1.41 to 6.23, *p* = 0.004, and HER2‐positive disease, adjusted hazard ratio 2.70, 95% CI 1.39 to 5.26, *p* = 0.003, were associated with poorer PFS relative to Luminal A.

**Conclusion:**

In this institution‐based cohort, molecular subtype remained an important determinant of progression‐related outcome. Luminal A showed the most favorable progression‐related profile, whereas Luminal B, HER2‐negative, and HER2‐positive disease had poorer PFS. The study also demonstrates active use of multimodality treatment and provides real‐world evidence on breast cancer care patterns at a tertiary oncology center in Bangladesh.

## 1 Introduction

Breast cancer remains a major cause of morbidity and mortality among women worldwide and continues to represent a substantial public health challenge. It accounts for approximately one quarter of all newly diagnosed cancers among women globally, with an estimated 2.3 million new cases and 685 000 deaths reported in 2020, according to GLOBOCAN data [1]. Despite major advances in screening, pathological classification, systemic therapy, and locoregional treatment, important disparities in breast cancer outcomes persist across regions. High‐income countries have achieved improved survival through organized screening programs, timely diagnosis, multidisciplinary care, and access to modern therapeutic options, whereas low‐ and middle‐income countries (LMICs) continue to face systemic barriers that adversely influence prognosis, including delayed presentation, limited diagnostic capacity, treatment interruptions, and unequal access to optimal care [2, 3].

In Bangladesh, breast cancer is the most frequently diagnosed malignancy among women, and its burden continues to increase. However, a substantial proportion of patients still present with clinically advanced disease, which remains a major determinant of poor outcomes. This pattern has been linked to multiple factors, including low awareness of early symptoms, sociocultural barriers to timely consultation, financial constraints, and the absence of a nationwide population‐based screening program [4]. In addition, access to specialized oncology services remains uneven, and breast cancer care is often delivered in a context shaped by resource limitations, variable referral pathways, and incomplete long‐term follow‐up. Prior reports from Bangladesh have highlighted delayed presentation, infrastructural limitations, and the shortage of specialized oncology personnel as continuing obstacles to improving cancer outcomes [5, 6]. Together, these factors create a clinical environment in which real‐world treatment delivery and outcome assessment may differ substantially from those observed in highly resourced settings.

Contemporary breast cancer management is inherently multimodal and relies on integration of surgery, chemotherapy, radiotherapy, endocrine therapy, and targeted therapy according to stage, tumor biology, and patient characteristics. In routine practice, treatment planning is also influenced by the availability of biomarker testing, the feasibility of breast‐conserving treatment, access to systemic agents, and the ability to maintain longitudinal follow‐up. In Bangladesh and other resource‐constrained settings, this process may be affected by delayed diagnosis, limited institutional capacity, incomplete documentation, and uneven access to specialized procedures and targeted agents. Earlier local reports have suggested that mastectomy has historically remained common and that breast‐conserving approaches have been less consistently adopted than in higher‐resource settings, reflecting both disease presentation and service‐level constraints [7]. Although neighboring countries in South Asia have taken steps toward more standardized cancer management through national or network‐based guidance, important differences in implementation remain. For example, India's National Cancer Grid has promoted context‐sensitive protocols for multimodal cancer care, while Sri Lanka has invested in public early‐detection and awareness structures that support more timely diagnosis in some settings [8, 9, 10]. These regional comparisons underscore the importance of institution‐level real‐world data in understanding how breast cancer is currently managed in Bangladesh.

Among the major determinants of treatment selection and prognosis in breast cancer, molecular subtype has particular clinical relevance. Classification into luminal A, luminal B, HER2‐enriched or HER2‐positive disease, and triple‐negative breast cancer provides a practical framework for anticipating treatment responsiveness and outcome differences [11, 12]. Luminal A tumors are generally associated with more favorable biology and responsiveness to endocrine therapy, whereas triple‐negative tumors typically exhibit more aggressive clinical behavior and poorer outcomes. Luminal B and HER2‐positive subtypes often occupy intermediate or high‐risk positions depending on proliferative index, receptor expression, and access to subtype‐specific therapy. Biomarkers such as estrogen receptor, progesterone receptor, HER2, and Ki67 are therefore important not only for subtype classification but also for guiding systemic treatment and refining prognostic assessment [13, 14]. In settings where access to molecularly guided treatment may be inconsistent, the relationship between subtype and outcome becomes particularly relevant for understanding patterns of care and identifying practical gaps in treatment delivery.

Clinical outcome measures, especially progression‐free survival (PFS) and overall survival (OS), are central to the evaluation of breast cancer treatment. PFS, commonly defined as the interval from initiation of treatment or study entry to progression or death, is frequently used in both clinical trials and real‐world analyses, especially in settings where long‐term death ascertainment is incomplete or delayed. Previous studies have demonstrated a meaningful association between PFS and OS in several breast cancer contexts, particularly in advanced disease, supporting the utility of progression‐based endpoints when interpreted carefully [15, 16]. At the same time, progression‐related outcomes can be difficult to measure consistently in LMIC settings because follow‐up systems may be fragmented, registries may be incomplete, and outcome documentation may vary across institutions and time periods. In Bangladesh, real‐world survival data remain limited, and there is no comprehensive public national cancer registry capable of routinely supporting longitudinal outcome evaluation across institutions. As a result, hospital‐based retrospective analyses remain an important source of evidence for understanding outcome patterns, treatment documentation, and survivorship trajectories in routine clinical practice.

Taken together, these considerations support the need for institution‐based analyses that describe how breast cancer is being managed within the care environment currently available in Bangladesh, particularly in relation to subtype distribution, treatment documentation, and progression‐related outcomes. The present study was therefore undertaken to examine the clinicopathological characteristics, molecular subtype patterns, documented treatment modalities, and survival‐related outcomes of breast cancer patients managed at a tertiary care center in Bangladesh through retrospective analysis of hospital records. By focusing on real‐world data from routine oncology practice, the study seeks to contribute context‐specific evidence on breast cancer management in Bangladesh while avoiding assumptions of national representativeness.

## 2 Methods

### 2.1 Study Design and Setting

This retrospective observational study was conducted at the Department of Oncology, Bangladesh Specialized Hospital, Dhaka, Bangladesh. The study duration was 1 year, from January 2024 to December 2024, and involved review of the medical records of breast cancer patients treated between 2012 and 2022. The study was based on routinely maintained hospital records of patients with breast cancer who were treated, evaluated, or followed up at this tertiary care center. In this study, the term “current standard of care” refers to the routine multimodality breast cancer management delivered at the study center during the study period, including combinations of surgery, chemotherapy, radiotherapy, endocrine therapy, and HER2‐directed therapy where clinically indicated.

### 2.2 Study Population

The study included adult patients with a documented diagnosis of breast cancer whose records contained sufficient clinical information for case‐level analysis. Patients were managed through routine oncology practice at Bangladesh Specialized Hospital, including both those whose treatment was initiated at the study center and those who presented after prior evaluation or treatment elsewhere for continuation of care, reassessment, recurrence management, or follow‐up. The final analytical cohort comprised 1058 patients.

### 2.3 Data Collection and Study Variables

Data were obtained from hospital medical records and associated clinical documentation. Extracted variables included demographic characteristics, clinicopathological features, treatment‐related information, and follow‐up data. Tumor‐related variables included histopathology, documented metastatic or recurrent disease during the available records, clinical stage at diagnosis, receptor status, and molecular subtype. Clinical stage referred to disease stage at diagnosis, whereas documented metastatic or recurrent disease referred to evidence of metastatic or recurrent disease identified at any time in the available records and was not restricted to initial presentation. Molecular subtype was categorized as Luminal A, Luminal B (HER2‐positive), Luminal B (HER2‐negative), HER2‐positive, triple‐negative breast cancer, or insufficient data when classification was not possible from the available record.

Treatment‐related variables included documented receipt of chemotherapy, endocrine therapy, breast surgery, HER2‐directed therapy, and radiotherapy. These treatment categories were analyzed as documented modalities and were not considered mutually exclusive. Breast surgery was further categorized as mastectomy, breast‐conserving surgery, or other breast surgery where specified in the record. Axillary surgery was classified according to the documented procedure, including axillary lymph node dissection and sentinel lymph node biopsy. Endocrine agents were summarized for luminal disease, and HER2‐directed agents were summarized for HER2‐positive disease.

### 2.4 Outcomes

The primary time‐to‐event outcome was PFS, defined as the interval in days from the analytical index date to the first documented disease progression or death from any cause, whichever occurred first. Patients without documented progression or death were censored at the date of last recorded follow‐up. OS was assessed descriptively from the available follow‐up records but was not used as the primary endpoint.

### 2.5 Statistical Analysis

Descriptive statistics were used to summarize demographic, clinicopathological, treatment, and outcome variables. Categorical variables were presented as frequencies and percentages. PFS was estimated using the Kaplan–Meier method overall and according to molecular subtype. Cox proportional hazards regression was used to estimate hazard ratios with 95% confidence intervals for PFS. Variables associated with PFS in univariate analysis were included in the multivariable model. Statistical analyses were performed using SPSS version 25.0, and a two‐sided *p*‐value of less than 0.05 was considered statistically significant.

### 2.6 Ethical Considerations

The study was conducted using retrospective hospital records, and all patient data were anonymized prior to analysis. Ethical approval was obtained from the institutional ethics committee of Bangladesh Specialized Hospital, reference number BSHL/Ethical Clearance/2023/09, approved on 19/03/2024. Individual informed consent was not applicable because this study was based solely on retrospective review of hospital records and involved no patient contact or intervention.

## 3 Results

As shown in Table 1, the cohort was predominantly female, 1045 (98.8%), and the largest age group was 50 to 59 years, 333 (31.5%). Overall, 377 patients (35.6%) were younger than 50 years. Hypertension, 128 (12.1%), and diabetes mellitus, 96 (9.1%), were the most frequently recorded comorbidities.

**TABLE 1:** Baseline demographic and treatment‐origin characteristics of the analytical cohort, *N* = 1058.

Characteristic	*n*	%
Age group		
< 30 years	11	1.0%
30 to 39 years	98	9.3%
40 to 49 years	268	25.3%
50 to 59 years	333	31.5%
60 to 69 years	262	24.8%
≥ 70 years	86	8.1%
Sex		
Female	1045	98.8%
Male	11	1.0%
Others	2	0.2%
Marital status (*n* = 1058)		
Married	916	86.6%
Unmarried	142	13.4%
Age of menarche (*n* = 1045)		
< 11	119	11.4%
11–15	867	83.0%
> 15	59	5.6%
Age of marriage (*n* = 916)		
< 18	210	22.9%
18–35	654	71.4%
> 35	52	5.7%
History of known risk factors (*n* = 1058)		
H/O smoking	12	1.1%
H/O HRT	43	4.1%
History of vontraception	15	1.4%
Family history of breast cancer	40	3.8%
Comorbidities (*n* = 1058)		
Bronchial asthma	21	2.0%
Hypertension (HTN)	128	12.1%
Diabetes mellitus (T2DM)	96	9.1%
Hypo/hyper thyroidism	31	2.9%
Chronic kidney disease (CKD)	1	0.1%
Ischemic heart disease (IHD)	14	1.3%
Others (unspecified/combined)	39	3.7%
No comorbidities	846	80.0%

As summarized in Table 2, Luminal A was the most common molecular subtype, 407 (38.47%), followed by TNBC, 249 (23.53%). Stage I disease was the largest stage group, 462 (43.66%), while Stage III and IV disease together accounted for 356 cases (33.65%). Invasive ductal carcinoma was the predominant histopathological type, 646 (61.06%).

**TABLE 2:** Clinicopathological summary of the analytical cohort.

Characteristic	*n*	%
Primary molecular subtype, *N* = 1058		
Luminal A	407	38.47%
Luminal B, HER2‐positive	102	9.64%
Luminal B, HER2‐negative	96	9.07%
HER2‐positive	126	11.91%
TNBC	249	23.53%
Insufficient data	78	7.37%
Documented metastatic or recurrent disease during available records, *N* = 1058		
Documented metastatic or recurrent disease present	366	34.59%
No documented metastatic or recurrent disease	692	65.41%
Clinical Staging at diagnosis (*N* = 1058)		
Stage IA	222	20.98%
Stage IB	240	22.68%
Stage IIA	144	13.61%
Stage IIB	96	9.07%
Stage IIIA	193	18.24%
Stage IIIB	144	13.61%
Stage IV	19	1.80%
Histopathology (*N* = 1058)		
Invasive ductal carcinoma	646	61.06%
Invasive lobular carcinoma	169	15.97%
Ductal carcinoma in situ	106	10.02%
Mucinous carcinoma	52	4.91%
Medullary Carcinoma	32	3.02%
Tubular carcinoma	21	1.98%
Papillary carcinoma	21	1.98%
Metaplastic carcinoma	11	1.04%

As shown in Table 3, chemotherapy was the most frequently documented treatment modality, 836 (79.02%), followed by breast surgery, 598 (56.52%), and endocrine therapy, 569 (53.78%). Among surgically documented patients, mastectomy was the most common procedure, 381 (63.71%). Among documented axillary procedures, ALND predominated, 389 of 416 (93.51%).

**TABLE 3:** Documented treatment modalities and multimodality overlap, *N* = 1058.

Treatment variable	*n*	%
Major treatment modalities, overlapping categories (*n* = 1058)		
Chemotherapy	836	79.02%
Endocrine therapy	569	53.78%
Breast surgery	598	56.52%
HER2‐directed therapy	224	21.17%
Radiotherapy documented	212	20.04%
Breast surgery (*n* = 598)		
Mastectomy	381	63.71%
Breast‐conserving surgery/lumpectomy	121	20.23%
Other	96	16.05%
Axillary surgery categories (*n* = 416)		
ALND	389	93.51%
SLNB	27	6.49%

Table 4 shows that endocrine therapy was documented in 524 of 605 luminal cases (86.61%), most commonly letrozole and tamoxifen. HER2‐directed therapy was documented in 196 of 228 HER2‐positive analytical cases (85.96%), with trastuzumab as the predominant agent.

**TABLE 4:** Endocrine and HER2‐directed agents in clinically relevant subtype‐defined groups.

Treatment agent	*n*	%
Luminal subgroup, *n* = 605		
Not documented	81	13.39%
Letrozole^a^	292	48.26%
Tamoxifen^a^	229	37.85%
Anastrozole^a^	24	3.97%
Fulvestrant^a^	20	3.31%
Leuprolide^a^	12	1.98%
Exemestane^a^	1	0.17%
HER2‐positive analytical subgroup, *n* = 228		
Not documented	32	14.04%
Trastuzumab	182	79.82%
Lapatinib^a^	20	8.77%
T‐DM1^a^	18	7.89%
Pertuzumab^a^	8	3.51%
Tucatinib^a^	6	2.63%

^a^ Overlapping treatment modalities were present for many patients.

As presented in Table 5, only 59 progression‐free survival events were recorded in the cohort, including 58 progression events and one death, while 999 patients (94.4%) were censored at last follow‐up.

**TABLE 5:** Progression‐free survival event summary in the analytical cohort, *N* = 1058.

PFS event category	*n*	%
Progression	58	5.5
Death	1	0.1
Censored	999	94.4

As shown in Table 6, the highest progression‐free survival event rates were observed in Luminal B HER2‐negative disease, 10.4%, and HER2‐positive disease, 9.5%, whereas Luminal A had a lower event rate of 4.2%.

**TABLE 6:** Distribution of progression‐free survival events by primary molecular subtype.

Primary subtype	*n*	PFS events, n	Event rate, %
Luminal A	407	17	4.2
Luminal B, HER2‐positive	102	6	5.9
Luminal B, HER2‐negative	96	10	10.4
HER2‐positive	126	12	9.5
TNBC	249	13	5.2
Insufficient data	78	1	1.3

**FIGURE 1:**
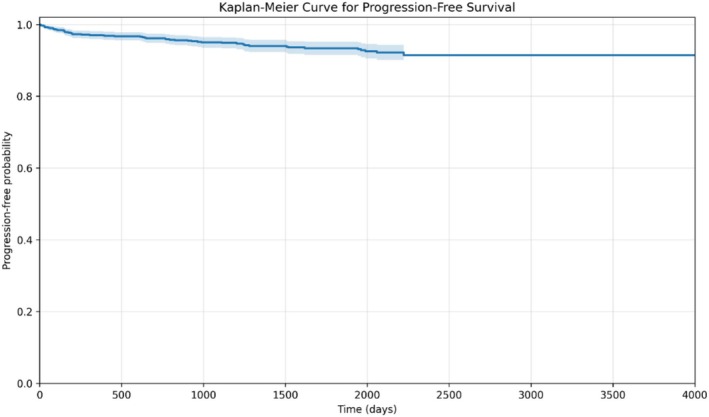
Overall Kaplan–Meier progression‐free survival curve with 95% confidence interval (*N* = 1058).

The overall Kaplan–Meier curve in Figure 1 shows a gradual decline in progression‐free survival over time, consistent with the low total number of recorded events.

**FIGURE 2:**
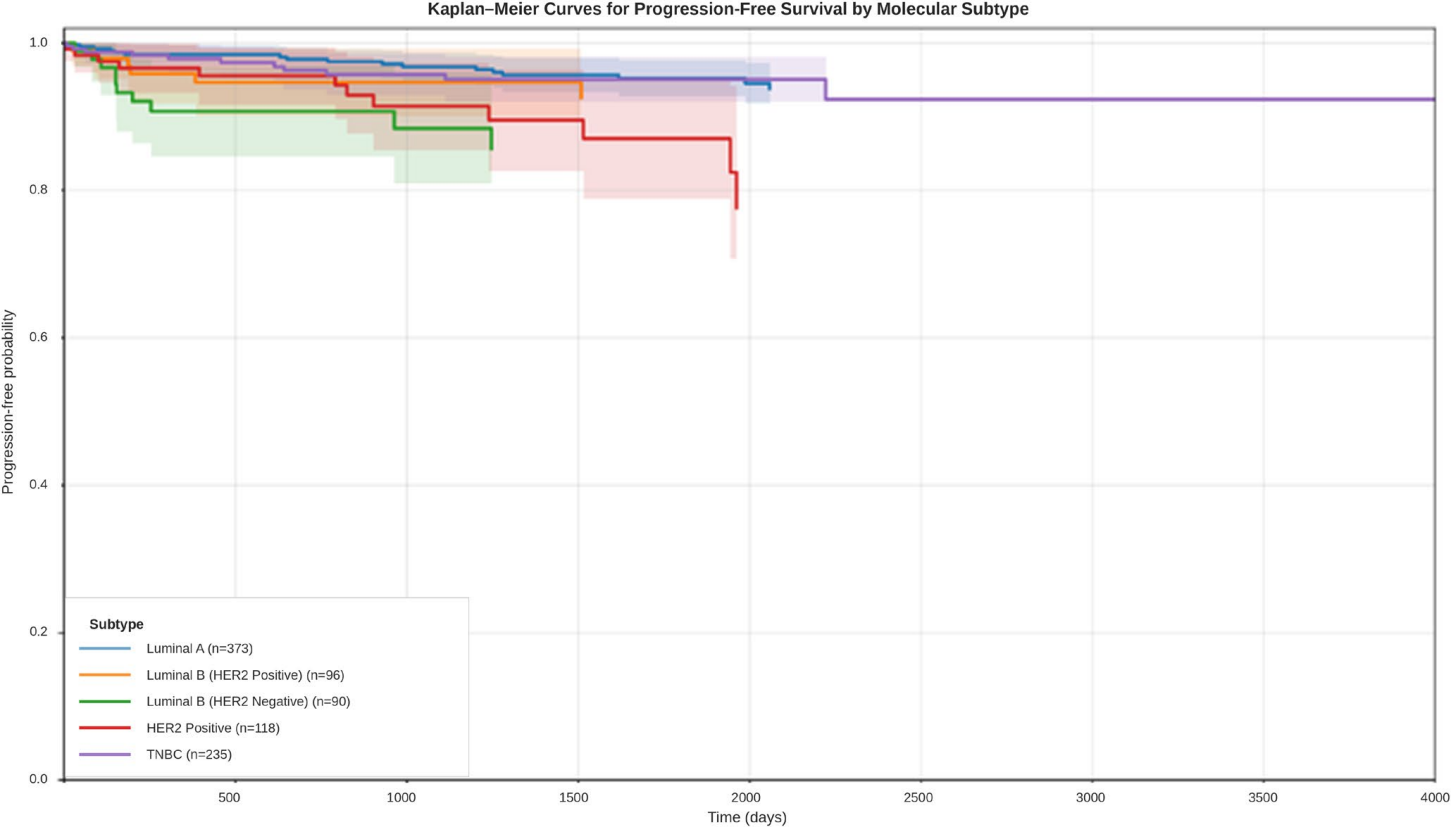
Kaplan–Meier progression‐free survival curves by primary molecular subtype (*n* = 1057).

As shown in Figure 2, Luminal A demonstrated the most favorable progression‐free survival pattern, whereas Luminal B HER2‐negative and HER2‐positive disease showed a greater decline over follow‐up.

In Table 7, Luminal B HER2‐negative disease, hazard ratio 3.39, and HER2‐positive disease, hazard ratio 3.19, were associated with significantly higher hazards of progression relative to Luminal A.

**TABLE 7:** Univariate Cox proportional hazards analysis for progression‐free survival.

Variable	Hazard ratio (HR)	95% confidence interval	*p*
Primary molecular subtype, reference = Luminal A			
Luminal B, HER2‐positive	1.64	0.64 to 4.20	0.300
Luminal B, HER2‐negative	3.39	1.49 to 7.70	**0.004**
HER2‐positive	3.19	1.50 to 6.78	**0.003**
TNBC	1.17	0.54 to 2.52	0.687
Age, per 1‐year increase	0.99	0.96 to 1.01	0.230
Documented metastatic or recurrent disease present	0.50	0.28 to 0.91	**0.023**

*Note:* *p*‐value of < 0.05 was considered statistically significant (in bold).

As shown in Table 8, Luminal B HER2‐negative disease, adjusted hazard ratio 2.97, and HER2‐positive disease, adjusted hazard ratio 2.70, remained independently associated with poorer PFS relative to Luminal A.

**TABLE 8:** Multivariable Cox proportional hazards analysis for progression‐free survival including variables significant in univariate analysis.

Variable	Adjusted hazard ratio (aHR)	95% confidence interval	*p*
Primary molecular subtype, reference = Luminal A			
Luminal B, HER2‐negative	2.97	1.41 to 6.23	**0.004**
HER2‐positive	2.70	1.39 to 5.26	**0.003**
Documented metastatic or recurrent disease present	0.52	0.29 to 0.94	**0.032**

*Note:* *p*‐value of < 0.05 was considered statistically significant (in bold).

## 4 Discussion

This single‐center, hospital‐based retrospective study provides real‐world evidence on breast cancer presentation, treatment documentation, and progression‐related outcomes in patients managed at a tertiary oncology center in Bangladesh. Three main patterns emerged from the analysis: Luminal A was the most common primary molecular subtype in the cohort, a substantial proportion of patients presented beyond early‐stage disease, and poorer progression‐free survival was observed in Luminal B HER2‐negative and HER2‐positive disease relative to Luminal A. These findings provide institution‐based insight into breast cancer care patterns within this tertiary oncology setting.

In the present cohort, Luminal A accounted for 38.47% of cases and TNBC for 23.53%, while invasive ductal carcinoma was the predominant histopathological type, accounting for 61.06% of tumors. This pattern is compatible with the established clinical importance of hormone receptor‐positive disease in breast cancer, while also indicating a substantial burden of biologically aggressive disease. A Bangladesh‐focused open‐access study by Islam, Islam, Dorin, and Jesmin reported meaningful variation in subtype distribution among Bangladeshi women and highlighted the importance of locally generated clinicopathological data when interpreting subtype burden in this setting [17]. The difference between that study and the present one should not be viewed as a contradiction, but rather as evidence that subtype distribution may vary according to institution, referral patterns, and cohort construction. Our findings therefore contribute center‐specific data that complement, rather than replace, the limited published literature from Bangladesh. In addition, 35.6% of patients in this cohort were younger than 50 years at diagnosis, which may reflect regional demographic structure, referral patterns, or healthcare‐seeking behavior within this institutional population.

Stage distribution in the present study also deserves emphasis. Stage I disease was the largest overall category, but Stages III and IV disease together still represented 33.65% of the cohort. In addition, documented metastatic or recurrent disease during the available records was present in 34.59% of patients. Because this latter variable was not restricted to disease status at initial diagnosis, it should not be interpreted as equivalent to Stage IV disease at presentation. These observations are consistent with prior Bangladeshi studies describing delayed diagnosis and advanced presentation as persistent challenges. In a tertiary hospital study from Dhaka, Akhtar, Hossain, Nahar, and Akhtar found that the mean duration required for diagnosis was 11 months and that patients commonly presented at Stages II, III, or IV, with none presenting at Stage I [18]. Similarly, Murshed, Hossain, Patwary, Roy, and Hussain reported that longer delays were associated with more advanced stage at presentation among Bangladeshi breast cancer patients [19]. Taken together, these comparisons suggest that although early‐stage disease is certainly represented in our cohort, late presentation remains a major clinical reality in routine practice.

The treatment findings are most appropriately interpreted as documentation of overlapping multimodality care rather than discrete treatment pathways. Chemotherapy was the most commonly documented modality, followed by breast surgery, endocrine therapy, HER2‐directed therapy, and radiotherapy. Among patients with documented breast surgery, mastectomy accounted for 63.71%, breast‐conserving surgery or lumpectomy for 20.23%, and other breast surgery for 16.05%. Among documented axillary procedures, ALND accounted for 93.51% and SLNB for 6.49%. These patterns suggest that extensive surgery remains common in this real‐world cohort. This observation is broadly consistent with the Bangladeshi experience reported by Akhtar et al. who showed that breast‐conserving treatment can be successfully delivered in selected patients, but also reflected the historical context in which breast‐sacrificing approaches remained common in local practice [7]. In our setting, the predominance of mastectomy and ALND likely reflects a combination of stage at presentation, advanced nodal disease, referral complexity, selective uptake of sentinel node procedures, surgeon preference, and practical service‐delivery constraints rather than a purely biology‐driven treatment pattern. Postoperative morbidity data, including outcomes such as lymphedema, were not uniformly documented in the available records and therefore could not be analyzed.

Among luminal cases, endocrine therapy was documented in 86.61%, with letrozole and tamoxifen being the most frequently recorded agents. Among the HER2‐positive analytical subgroup, HER2‐directed therapy was documented in 85.96%, and trastuzumab was the predominant recorded agent. These findings are clinically important, because real‐world access to prolonged endocrine treatment and HER2‐targeted therapy is often constrained in lower‐resource settings. A recent open‐access study from Botswana found multilevel barriers to accessing and adhering to endocrine therapy among breast cancer survivors, underscoring the gap between indication and sustained treatment use in routine practice [20]. Economic analysis from sub‐Saharan Africa has similarly shown that trastuzumab poses major affordability challenges in lower‐resource health systems, even when clinically indicated [21]. Against that background, the treatment documentation in the present cohort suggests that subtype‐appropriate systemic therapy was recorded for a substantial majority of eligible patients at this center, although documentation of receipt should not be interpreted as proof of uninterrupted access, adherence, or full‐course completion.

The survival findings should be interpreted within the framework of progression‐free survival, not OS. In the present cohort, only 59 total PFS events were observed, comprising 58 progression events and one death, while 999 patients were censored. Accordingly, the overall Kaplan–Meier curve showed only a gradual decline, and the subtype‐stratified curves demonstrated modest rather than dramatic separation. Even within this relatively low‐event setting, however, subtype remained prognostically informative. In univariate analysis, Luminal B, HER2‐negative, and HER2‐positive disease had significantly higher hazards of progression than Luminal A, and both associations remained significant in multivariable analysis. This direction of effect is consistent with the wider literature. Haque et al. showed that breast cancer subtype meaningfully influences survival across long‐term follow‐up, with less favorable outcomes in higher‐risk non‐Luminal A groups [22]. Fallahpour, Navaneelan, De, and Borgo likewise demonstrated survival heterogeneity across molecular subtypes in a population‐based registry analysis [23]. In addition, Lian, Fu, Chen, and Wang reported that prognosis varies by subtype and nodal context, with less favorable patterns in Luminal B and HER2‐related disease than in Luminal A [24]. Our findings therefore support the continued prognostic value of molecular subtype in routine institutional practice.

At the same time, the results should not be overinterpreted. TNBC did not show a statistically significant adverse association with PFS in the present Cox models, despite its recognized clinical aggressiveness. This should not be taken to mean that TNBC behaved favorably in a biological sense. A more plausible explanation is that the combination of low‐event counts, limited death ascertainment, retrospective follow‐up structure, and event distribution across groups reduced statistical separation for TNBC in this dataset. This interpretation is more consistent with the broader subtype‐survival literature, which generally identifies TNBC as a poorer‐prognosis category than Luminal A [22, 23]. The findings support several practical priorities for strengthening breast cancer care in similar institutional settings. Efforts to promote earlier diagnosis and timelier referral may help reduce the burden of advanced‐stage presentation. Continued strengthening of biomarker‐based classification and subtype‐guided treatment planning is also important, given the observed prognostic differences across molecular subtypes. Surgical practice may benefit from expanded multidisciplinary coordination and greater adoption of breast‐conserving and sentinel node approaches where clinically appropriate. Finally, more structured longitudinal follow‐up and outcome documentation would improve the quality of future real‐world survival analyses.

### 4.1 Strengths and Limitations

This study provides real‐world institutional evidence from a relatively large cohort of 1058 breast cancer patients managed at a tertiary oncology center in Bangladesh. It describes molecular subtype distribution, documented multimodality treatment patterns, and progression‐related outcomes using PFS as the primary time‐to‐event endpoint. The study also reports endocrine therapy and HER2‐directed therapy within clinically relevant subtype groups, which adds practical detail to the treatment profile of this cohort.

Several limitations should be considered. This was a retrospective single‐center study, so the findings are not nationally representative. Treatment variables reflected documented receipt rather than standardized information on treatment completion, adherence, toxicity, or postoperative morbidity. In addition, the survival analysis was based on a relatively small number of events, with a high proportion of censored observations, which limits the precision of PFS estimates. The variable describing documented metastatic or recurrent disease reflected evidence recorded at any time in the available records and was not restricted to baseline disease status alone, which should be considered when interpreting its relationship with clinical stage and survival outcomes. A stage‐ or metastatic‐status‐stratified survival analysis was not performed because the total number of progression‐free survival events was limited, and further subclassification under these conditions could have produced unstable or clinically misleading estimates.

## 5 Conclusion

This hospital‐based retrospective study provides real‐world evidence on breast cancer presentation, treatment documentation, and progression‐related outcomes at a tertiary oncology center in Bangladesh. Luminal A was the most common molecular subtype and showed the most favorable progression‐related profile, whereas Luminal B, HER2‐negative, and HER2‐positive disease were associated with poorer PFS relative to Luminal A. The treatment data indicate active use of multimodality care, with chemotherapy being the most frequently documented modality and with endocrine therapy and HER2‐directed therapy documented in a substantial proportion of clinically relevant subtype groups. Among surgically documented cases, mastectomy and axillary lymph node dissection were more common than breast‐conserving surgery and sentinel lymph node biopsy. Overall, these findings support the clinical relevance of molecular subtype in routine practice and contribute institution‐based evidence on breast cancer care in Bangladesh.

## Author Contributions

**Md Nurunnabi:** data curation, resources. **Ayasha Shiddika:** data curation, writing – original draft. **Salma Sultana:** investigation, writing – review and editing. **Md Arifur Rahman:** conceptualization, writing – original draft, methodology, project administration, supervision, data curation, investigation. **Qamruzzaman Chowdhury:** conceptualization, writing – review and editing, data curation. **Mashud Parvez:** data curation, software, formal analysis, writing – review and editing, visualization, validation. **Sharmin Akter Rupa:** investigation, data curation. **Ferdous Ara Begum:** data curation, methodology, formal analysis. **S. M. Khodeza Nahar Begum:** formal analysis, validation.

## Funding

The Contract Research Organization (CRO) was provided unconditional funding by Novartis (Bangladesh) Limited for data analysis and publication associated with this study while no funds were provided to the healthcare organization (HCO).

## Conflicts of Interest

The authors declare no conflicts of interest.

## Data Availability

The datasets used and/or analyzed during the current study are not publicly available because they were derived from hospital medical records containing potentially identifiable patient information. De‐identified data may be made available from the corresponding author on reasonable request, subject to institutional permission and applicable ethical considerations.
